# A review on modafinil: the characteristics, function, and use in critical care

**DOI:** 10.1080/21556660.2020.1745209

**Published:** 2020-04-04

**Authors:** Seyed MohammadReza Hashemian, Tayebeh Farhadi

**Affiliations:** a Chronic Respiratory Diseases Research Center (CRDRC), National Research Institute of Tuberculosis and Lung Diseases (NRITLD, Shahid Beheshti University of Medical Sciences, Tehran, Iran; b Clinical Tuberculosis and Epidemiology Research Center, National Research Institute of Tuberculosis and Lung Disease (NRITLD), Shahid Beheshti University of Medical Sciences, Tehran, Iran

**Keywords:** modafinil, critically ill patients, narcolepsy, sleep disorders

## Abstract

In critically ill patients, sleep is generally interrupted. Some factors that lead to such sleep interruption include the intensive care unit (ICU) circumstance, primary medical disease itself, mental stress, and impacts of many drugs and other managements utilized to treat ICU patients. Another illness that may cause profound daytime somnolence is narcolepsy. Modafinil, methylphenidate and amphetamines are used as stimulants to treat symptoms, such as extreme daytime sleepiness, cataplexy and nocturnal sleep disruption. Such stimulants can increase awareness, improve perception and thinking, as well as assist in keeping people awake. The exact mechanism of action of modafinil is unclear. *In vitro* studies have demonstrated that binding of modafinil to the dopamine reuptake pump can prevent the reuptake of dopamine, resulting in a boost in extracellular dopamine. Modafinil is a racemic compound containing *l* and *d* isomers. Peak plasma concentrations of the drug occur at 2–4 h after administration; therefore, the absorption of modafinil is considered fast. Modafinil is properly distributed in tissues by binding to plasma proteins moderately. Despite the likely role of modafinil in improving cognition and arousal in critically ill patients, the available data on the use of modafinil in the ICU setting is limited. The aim of the study was to review the novel usage of modafinil for alleviation of fatigue, excessive daytime somnolence (EDS), and/or depression in critically ill patients.

## Introduction

In critically ill patients, sleep is generally interrupted. Some factors that lead to such sleep interruption include the intensive care unit (ICU) circumstance, primary medical disease itself, mental stress, and impacts of many drugs and other managements utilized to treat ICU patients^1^.

Another illness that may cause profound daytime somnolence is narcolepsy. Narcolepsy is not equal to somnolence. It is a particular chronic neurologic disease with a strong HLA association. Narcolepsy is a multifactorial disease, and genetic differences at multiple loci are related to the disease. Genome-wide association studies showed more than ten genomic variations are related to narcolepsy. Moreover, DNA genome sequencing revealed rare variants related to narcolepsy^2^. Nearly all patients will narcolepsy have particular HLA markers including HLA DRB1 05:01 and DQB1 06:02^1^.

Patients with narcolepsy will sometimes become patients in ICU. Untreated narcolepsy may result in somnolence in the patients and can be an unknown reason of reduced psychological status in the critically ill patients. Cataplexy is a medical condition in which emotions such as laughing, crying, or terror cause a patient undergo sudden and transient episodes of muscle weakness accompanied though remaining conscious awareness^3^. Cataplexy affects near 70 percent of patients with narcolepsy^2^^,^^4^. In such patients, there is an autoimmune destruction in the hypothalamic neurons producing the neuropeptide hypocretin (orexin). Orexin regulates arousal and play a role in balance of the transition between sleep and wake^4^.

At present, there is symptomatic treatment for narcolepsy patients, although there is no cure for the condition. Modafinil, methylphenidate and amphetamines are used as stimulants to treat symptoms such as extreme daytime sleepiness, cataplexy and nocturnal sleep disruption. Modafinil is indicated to improve wakefulness in patients with excessive daytime sleepiness associated with narcolepsy (https://www.drugbank.ca/drugs/DB00745). Methylphenidate is indicated to treat attention deficit hyperactivity disorder in patients 6 years of age and older and for the treatment of narcolepsy (https://www.drugbank.ca/drugs/DB00422). Amphetamine is indicated for the treatment of attention-deficit/hyperactivity disorders as well as for the treatment of central nervous system disorders such as narcolepsy (https://www.drugbank.ca/drugs/DB00182). Other therapeutics to treat the symptoms are tricyclic antidepressants, serotonin-specific reuptake inhibitors and sedative-hypnotics (sodium oxybate)^5^.

**Figure 1.**
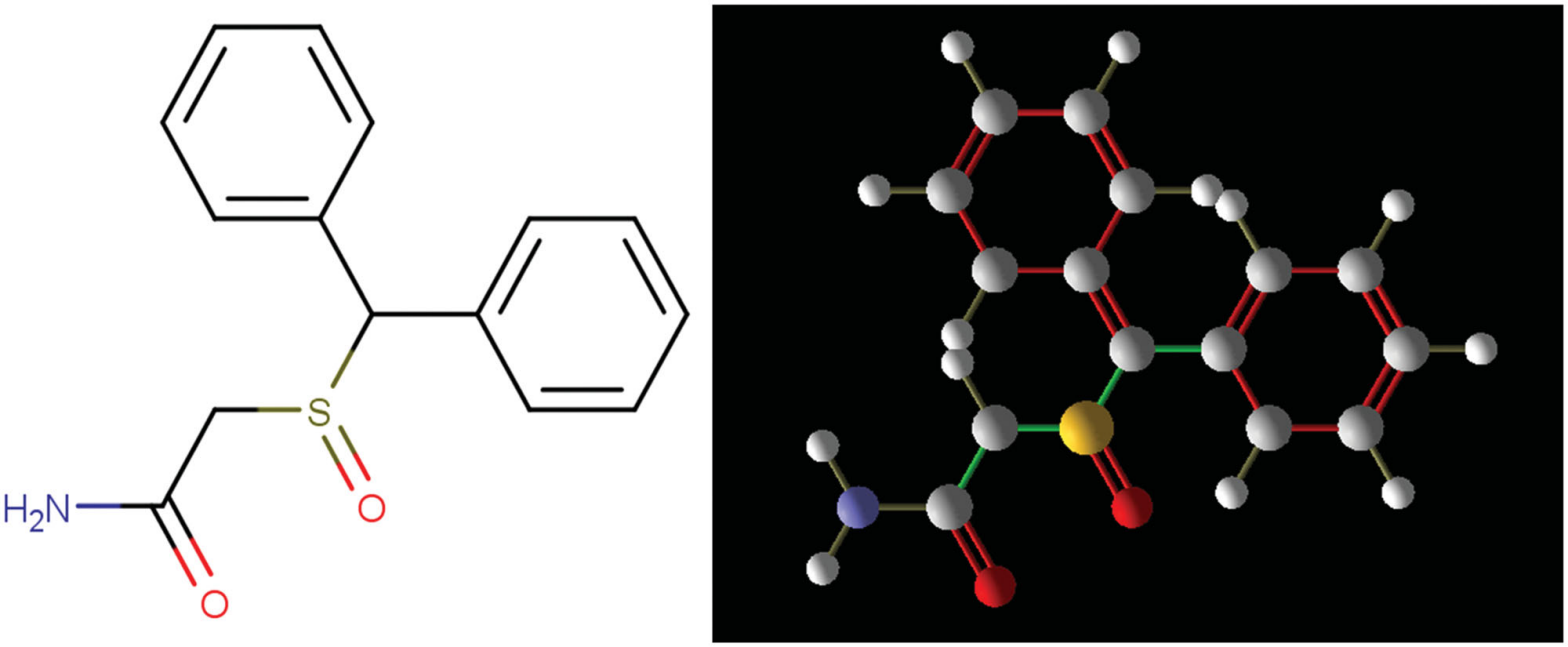
Chemical structure of modafinil. The empirical formula of the drug is C_15_H_15_NO_2_S (molecular weight: 273.35 g/mol).

As mentioned above, modafinil is a wakefulness-promoting medication used to treat excessive daytime sleepiness. It has been revealed that modafinil decreases fatigue and betters quality of life for individuals with narcolepsy^6^^,^^7^. Chemical structure of modafinil is shown in Figure 1.

Despite the likely role of modafinil in improving cognition and arousal in critically ill patients, the available data on the use of modafinil in the ICU setting is limited. The aim of the study was to review the novel usage of modafinil for alleviation of fatigue, excessive daytime somnolence (EDS), and/or depression in critically ill patients.

## Mechanism of action of modafinil

The exact mechanism of action of modafinil is unclear. It has been revealed that modafinil has neuroprotective and antioxidative effects, which have not formerly been proposed to be associated with its wake-promoting properties. Recently, researchers have indicated that free radicals may be associated with sleep stimulation as well as cellular destruction, signifying that modafinil may have a common target of action to oppose both of these outcomes^6^^,^^7^.

*In vitro* studies have demonstrated that binding of modafinil to the dopamine dopamine transporter in the striatum can prevent of the reuptake of dopamine resulting in a boost in extracellular dopamine. Modafinil stimulates glutamatergic circuits while preventing gamma-aminobutyric acid (GABA).

Comparing to other stimulants, modafinil has shown low potential for abuse since it has not important pleasurable or euphoric properties. A synergistic combination of mechanisms including direct prevention of dopamine reuptake, indirect prevention of noradrenalin reuptake in the ventrolateral preoptic (VLPO) nucleus and orexin activation has been attributed to modafinil. Besides, modafinil has partial alpha 1-adrenergic agonist properties by directly inducing the receptors^6–8^.

## Pharmacokinetics

Modafinil is a racemic compound containing *l* and *d* isomers. The enantiomers have dissimilar pharmacokinetics and do not interconvert. In adult humans, the half-life of the *l*-isomer is about three times that of the *d-*isomer and consequently, overall exposure to the *l-*isomer is almost three times that for the *d*-isomer. After once daily dosing of modafinil, the *l*-isomer and the *d*-isomer form 90 percent and 10 percent of the concentration of the circulating drug, respectively. After multiple doses, the efficient elimination half-life of the drug is approximately 15 h^9^.

Peak plasma concentrations of the drug occur at 2–4 h after administration, therefore, the absorption of modafinil is considered to be fast. The bioavailability of modafinil is almost equivalent to that of a liquid suspension. The total oral bioavailability was not found out because of the water insolubility (<1 mg/mL) of modafinil, which prevented intravenous application. Nourishment has no impact on general modafinil bioavailability; yet, its absorption may be postponed by nearly one hour if taken with food^9^.

Modafinil is properly distributed in tissues by binding to plasma proteins moderately. The volume of distribution (∼0.9 L/kg) of modafinil is obviously greater than the volume of total body water (0.6 L/kg). It is illustrated that modafinil is moderately bound to plasma protein *in vitro* (∼60%, essentially to albumin). Modafinil displays no dislocation of protein binding of diazepam, warfarin or propranolol at serum concentrations <500μM. At concentrations >500μM, modafinil acid reduces the degree of warfarin binding, but these concentrations are >35 times those obtained therapeutically^9^.

The main way of elimination of modafinil is metabolism by the liver (90 percent), with following renal removal of the metabolites. Chemical reactions involved in the compound metabolism include hydrolytic deamidation, S-oxidation, aromatic ring hydroxylation, and glucuronide conjugation. The original molecules form less than 10 percent of the excretion of an administered dose^9^.

## Side effects of modafinil

The main side effects related to modafinil include nausea and headache. Research studies and user reviews have reported headaches as most prevalent side effect that may occur in over 35 percent of patients who take modafinil. Diarrhea, nose and throat congestion, back pain, dry mouth, anxiety, nervousness, insomnia, dizziness and mental side effects are mild side effects that have been described^10^.

In some cases, by first start taking of modafinil, side effects may occur. In cases where an adjustment period is required, side effects often reduce after a few weeks since the body adjusts to the agent. The incidence of side effects may be simply because of dosage level. In some cases, reducing the dosage to a manageable level can assist to remove or decrease side effects.

In some people that have a genetic predisposition, a severe rare disorder in skin and mucous membrane including Stevens-Johnson syndrome, toxic epidermal necrolysis (TEN), or drug reaction with eosinophilia and systemic symptoms (DRESS) may be developed by using modafinil. The evidence about this disorder is not completely conclusive at this time, but it is significant to be conscious of the possibility^10^.

## Drug-drug interactions

In liver, 3 A isoform subfamily of hepatic cytochrome P450 (CYP3A4) metabolizes a high percentage of modafinil. However, modafinil is potent to suppress CYP2C19 and CYP2C9, and is able to induce CYP3A4, CYP1A2 and CYP2B6^9^.

CYP2C19 is a drug-metabolizing enzyme and involves in elimination of diazepam, phenytoin and propranolol. Therefore, co-administration of modafinil with the mentioned drugs, may increase the circulating levels of those compounds^9^.

It is reported that 7–10 percent of the Caucasian population (similar or lower in other populations) are defective in the enzyme CYP2D6. In such individual, by co-administration of modafinil, the levels of CYP2D6 substrates (e.g. selective serotonin reuptake inhibitors and tricyclic antidepressants) which have supplementary pathways of elimination *via* CYP2C19, may be heightened. According to, while co-administration of modafinil with the mentioned therapeutics, it is necessary to adjust the drugs dosages^9^.

Coadministration of other central nervous system (CNS) active therapeutics such as dextroamphetamin and methylphenidate with modafinil do not meaningfully change the pharmacokinetics of either medicine^9^.

It has been found that chronic administration of modafinil 400 mg can induce CYP3A4 and consequently decrease the systemic exposure to CYP3A4 substrates such as ethinyl estradiol and triazolam. Chronic administration of modafinil can enhance the removal of CYP3A4substrates. Dose adjustments may be required for individuals being treated with these and similar drugs^9^.

After exposure to modafinil *in vitro*, an obvious concentration-associated inhibition of CYP2C9 functionality was seen in human hepatocytes. It demonstrates that there is a possibility for a metabolic interaction between modafinil and the substrates of this enzyme such as S-warfarin and phenytoin. However, an interaction survey in healthy volunteers reveled that chronic modafinil treatment do not significantly influence on the pharmacokinetics of warfarin when compared to placebo^9^.

## Modafinil tolerance

Much of the research on the topic of modafinil have shown that modafinil is well tolerated^11–13^. For example, one study was conducted on depressed patients using modafinil to relieve symptoms of depression, including fatigue and sleepiness. The patients did not show risking a switch in their mood or developing tolerance or abuse of modfinil during two months of treatment^11^.

Some studies were conducted to survey the possibility of modafinil tolerance in pediatric patients that used the drug for narcolepsy. The results showed that no significant number of the patients developed tolerance^14^.

However, there are some evidences about induction of modafinil tolerance in some patients by long-term use of the drug^15–18^. This may cause the patient need higher daily doses in order to reach the same cognitive enhancement advantages or the same level of relief from sleepiness problems. One approach to avoid the potential risk of developing modafinil tolerance issues is to skip a dose of the drug at one or even two days of a week.

Patients with present or past addictions to substances such as alcohol, cocaine, or nicotine and individuals with a family history of addiction are at highest risk of drug tolerance^15–16^.

A number of mechanisms may involve in inducing tolerance to modafinil. First, it has been reported that modafinil blocks dopamine transporter in animals and human brain resulted in inhibition of dopamine reuptake^17^. Inhibition of dopamine reuptake increases the extracellular levels of dopamine in the brain leading to disrupting wake-promoting actions in knock-out mice^17^. Blocking the dopamine reuptake may increase dopamine in the nucleus accumbens and can be connected to the drug abuse. This issue emphasizes the avoidance of the long-term use of modafinil^17^.

In addition to dopamine, as one of the main chemicals influencing modafinil tolerance, modafinil can change norepinephrine levels in the brain, but not by adequate to induce addiction or euphoria^17^.

The precise mechanism of modafinil tolerance is not completely recognized, however, there is some anecdotal evidence that can assist one to better comprehend modafinil tolerance and how to avoid it.

## The use of modafinil in ICU

In adults with narcolepsy (non-critically ill patients), it was shown that modafinil (200–400 mg/day) could recover daytime somnolence, increase scores on Clinical Global Improvement of Change (CGI-C), and enhance mean sleep latencies on maintenance of wakefulness test (MWT)^13^^,^^19–21^. In narcolepsy patients, split dosing (200 mg at 7 am and 200 mg at noon) was shown to be more efficient than a single dose in the morning; 600 mg/day split dosing may be required by some^19^. In narcolepsy patients with residual evening sleepiness, somnolence was significantly improved in 80 percent and 92 percent of the drug users at doses of 400 and 600 mg, respectively^19^. Compared to modafinil, armodafinil may have a longer duration of activity^22^.

In ICU, the focus of critical care becomes patients’ rehabilitation as critically ill patients recover from their disease. The patient recovery may be delayed and outcomes may potentially worse due to fatigue, excessive daytime somnolence (EDS), and depression^23^. Psychostimulants, specifically modafinil alleviated some of these signs in different patient populations. Clinical trials are underway discovering this novel usage of the medicine^23^. A study on a case series of three patients in an institution’s Thoracic Surgery ICU was conducted^23^. The patients were selected as a result of their fatigue, EDS, and/or depression, and were given 200 mg of modafinil each morning to improve patient wakefulness, encourage their participation, and permit a more restful sleep during the night. Results showed that modafinil has potential advantages when used in some critically ill patients to accelerate patient recovery and shorten ICU stay^23^.

Although modafinil therapy has the potential to speed up patient recovery and reduce ICU stay, but there are few data on modafinil utilizing in the ICU setting. In a retrospective cohort study, the role of modafinil for improvement in cognition was surveyed in critically ill patients. Totally, 60 ICU patients with any ventilatory support who started on modafinil during their ICU stay were assessed. The average daily modafinil dose of 170 mg was given for a median duration of 9 days. After controlling for baseline severity of disease, age, and changes in sedation and analgesia over time, modafinil administration was seen to be associated with a small and non-significant increase in average scores of the Glasgow Coma Scale (GCS) by 0.34 points. No important modafinil-related side effects were detected. Modafinil administration did not meaningfully increase cognitive function in critically ill patients within 48 h of initiation. However, because of absence of strong data, the influence of modafinil on general patient outcomes in the ICU remains ambiguous and requires more examination^24^.

Modafinil has displayed slight probability for dependence or abuse, and it does not seem to interrupt usual sleep architecture^24^. Because of appropriate safety profile and absence of drug interactions, modafinil has been considered a promising drug for utilize in ICU patients^24^.

Because of a number of factors such as medications, the type of critical illness, substance abuse, oversedation and pain, critically ill patients are sensitive to develop neurocognitive problems^25^. It is proposed that the promptly and active mobilization of patients in the ICU importantly diminishes the possibility and period of delirium and can lead to reducing ICU length of stay^26–28^. Critically ill patients who are capable to obtain wakefulness are more anticipated to involve in aggressive physical therapy^29–30^. Gajewski et al. reported that they observed clinical improvement after the beginning of modafinil treatment in 3 non-intubated critically ill patients who were not receiving sedatives^23^. Despite of the likely role of modafinil in improving cognition and arousal in critically ill patients, the available data on the use of modafinil in the ICU setting is limited.

## Transparency

### Declaration of financial/other relationships

The authors and peer reviewers on this manuscript have no relevant financial or other relationships to disclose.
